# Open Conformation of the *Escherichia coli* Periplasmic Murein Tripeptide Binding Protein, MppA, at High Resolution

**DOI:** 10.3390/biology7020030

**Published:** 2018-05-19

**Authors:** Forum Bhatt, Vishal Patel, Constance J. Jeffery

**Affiliations:** Department of Biological Sciences, MC567, University of Illinois, 900 S. Ashland Ave, Chicago, IL 60607, USA; forumbf@yahoo.com (F.B.); vpatel823@gmail.com (V.P.)

**Keywords:** periplasmic ligand-binding protein, murein tripeptide, murein recycling, ligand binding, transmembrane transport

## Abstract

Periplasmic ligand-binding proteins (PBPs) bind ligands with a high affinity and specificity. They undergo a large conformational change upon ligand binding, and they have a robust protein fold. These physical features have made them ideal candidates for use in protein engineering projects to develop novel biosensors and signaling molecules. The *Escherichia coli* MppA (murein peptide permease A) PBP binds the murein tripeptide, l-alanyl-γ-d-glutamyl-meso-diaminopimelate, (l-Ala-γ-d-Glu-meso-Dap), which contains both a D-amino acid and a gamma linkage between two of the amino acids. We have solved a high-resolution X-ray crystal structure of *E. coli* MppA at 1.5 Å resolution in the unliganded, open conformation. Now, structures are available for this member of the PBP protein family in both the liganded/closed form and the unliganded/open form.

## 1. Introduction

Periplasmic ligand-binding proteins (PBP) of Gram-negative bacteria and the homologous membrane-bound lipoproteins of Gram-positive bacteria, along with their cognate membrane-embedded permeases, constitute a large class of active transport systems that are responsible for the uptake of sugars, amino acids, anions, peptides, and other nutrients [1]. The PBPs have a high specificity and affinity for their ligands, with Kds in the range of 0.1 μM for amino acids and 1 μM for sugars, and they are the major determinants of transporter specificity [2]. Some of the periplasmic binding proteins also play a role in bacterial chemotaxis by binding to inner membrane receptors.

The three-dimensional X-ray crystal structures of dozens of PBPs have been determined with and without ligands bound. Structures include those with a specificity towards various sugars including arabinose [3], galactose [4], ribose [5], and maltose [6], anions including sulfate [7] and phosphate [8], amino acids including histidine [9,10], leucine [11], leucine/isoleucine/valine [12], and lysine/arginine/ornithine [13], oligopeptides [14,15], dipeptides [16,17], and metal ions, including nickel [18].

Although they do not share a significant amino acid sequence identity, members of the PBP family share a bilobed structure with two relatively rigid domains connected by a hinge region that facilitates their movement relative to each other. In the absence of ligands, the protein is in an extended or open conformation. The ligand binds in a deep cleft between the two lobes, bringing the two lobes together to enclose the ligand in a mechanism similar to a Venus fly trap [19]. Although the open form of the binding proteins exposes a solvent accessible cleft, the closed form encloses the bound ligand in a protected protein interior completely inaccessible to the bulk solvent. This conformational change upon ligand binding is essential for the recognition of the PBPs by their respective membrane-bound components because it is the ligand-bound closed form that is recognized by transporters and chemotaxis receptors. Residues that interact with the transporter or the receptor cluster in patches on the two lobes of the binding protein. The patches are brought near each other due to the conformational change that occurs when the ligand binds [20]. Once bound to the cognate transmembrane transporter, the PBP releases its ligand, and the ligand crosses the inner membrane through the transporter. 

Due to the large diversity of the proteins in this family and the high affinity for their ligands, the members of this family have provided an exceptional source for understanding the mechanisms of ligand binding and transport. In addition to their ligand affinity and specificity, the large conformational change upon ligand binding and the significant stability of the PBP fold have made them ideal candidates for redesign and usage as novel biosensors and signaling molecules (reviewed in [21,22,23]).

The binding proteins range in size from 20–60 kD, with the peptide binding proteins being the largest of all and binding some of the largest substrates transported by this protein family. In Gram-negative bacteria, the oligopeptide binding protein (OppA) recognizes peptides that are three to five residues long, while the dipeptide binding protein (DppA) recognizes peptides two residues long, and in Gram-positive bacteria, the oligopeptide binding protein AppA recognizes peptides that are two to nine residues long. Another ligand binding protein of this subgroup, *Escherichia coli* MppA (murein peptide permease A), is required for the uptake of the murein tripeptide, l-alanyl-γ-d-glutamyl-*meso*-diaminopimelate, (l-Ala-γ-d-Glu-*meso*-Dap), which is generated by the breakdown of the cell wall murein from the periplasm in the recycling of cell wall peptides [24]. This unusual peptide contains an L-Ala linked to a D-Glu, and the D-Glu has a γ linkage to *meso*-Dap. MppA binds the peptide with high affinity, with a Kd of 250 nM [25]. MppA is a 58 kDa protein that shares an overall amino acid sequence identity of 46% with the oligopeptide binding protein (OppA) and a 29% identity with the dipeptide binding protein (DppA). It is not found in its own transport operon but uses the oligopeptide permease or the dipeptide permease for the transmembrane transport of its ligand [26].

The crystal structure of MppA with the bound murein tripeptide has been reported [25]. In order to gain further insight into the conformational changes associated with ligand binding and complete the structural information for this protein, we report here a high-resolution structure of unliganded *E. coli* MppA, the murein tripeptide binding protein, at a resolution of 1.5 Å.

## 2. Materials and Methods

### 2.1. Expression of 6-His Tagged MppA

*E. coli* strain JM109 harboring the plasmid pQE60 [27], which encodes MppA with a C-terminal 6-histidine tag, was a kind gift from Dr. James Park (Tufts New England Medical Center). 2xYT broth with 50 µg/mL ampicillin was inoculated with overnight cultures (1:20 dilution) and grown at 37 °C until the cells reached an OD_600_ of 0.4. MppA expression was then induced with 1.0 mM isopropyl-β-d-thiogalactopyranoside (IPTG). The cultures were grown for four more hours at 25 °C and then harvested by centrifugation. The cell pellet was washed once in 100 mM Tris-HCl, pH 7.6, and frozen at −80 °C until further use.

### 2.2. Purification of MppA

The cells were resuspended in 100 mM Tris-HCl, pH 7.6 (binding buffer), and lysed by sonication. The cell suspension was centrifuged at 10,000× *g* for 30 min to remove cell debris. The cleared lysate was incubated for two hours with gentle agitation at 4 °C with Ni-NTA beads (Qiagen, Germantown, MD, USA) that were pre-equilibrated with a binding buffer. The beads were washed with eighty column volumes of binding buffer, followed by ten column volumes of 0.3 M NaCl, 0.1 M KHPO_4_, pH 7.0, 10 mM imidazole, and 5% glycerol. The protein was eluted with five column volumes of 0.3 M NaCl, 0.1 M KHPO_4_, pH 7.0, 0.5 M imidazole, and 5% glycerol. The buffer was then changed to 100 mM Tris-HCl, pH 7.6, by dialysis. This was followed by an ammonium sulfate precipitation step in which 55% ammonium sulfate saturation was obtained by gradually adding solid ammonium sulfate with constant stirring on ice. The resulting solution was centrifuged at 12,000 rpm (17211× *g*) for 30 min, and the pellet was discarded. Additional ammonium sulfate was added to the supernatant to achieve 80% saturation, and the solution was centrifuged again. MppA was obtained in the pellet fraction. The pellet was resuspended in 100 mM Tris-HCl, pH 7.6, and dialyzed overnight against the same buffer to remove the ammonium sulfate. This fraction, which contained almost pure MppA, was concentrated by ultrafiltration to 10 mg/mL concentration by using spin concentrators. The protein was visualized by SDS–PAGE on a 12% polyacrylamide gel.

### 2.3. Crystallization of MppA and X-Ray Data Collection

Initial crystallization conditions were identified through sparse matrix sampling [28]. Crystals with dimensions 0.1 × 0.1 × 0.3 mm were obtained in one to three weeks by micro- or macro-seeding in a hanging drop. The drop contained 2 µL of 10 mg/mL protein solution and 2 µL of the reservoir solution containing 0.2 M sodium acetate trihydrate, 0.1 M Tris-HCl, pH 8.5, and 30% *w/v* PEG 4000, (Crystal Screen I, solution #22, Hampton Research, Aliso Viejo, CA, USA). MppA crystallizes in space group P2_1_. X-ray diffraction data were collected using a Mar CCD detector (Rayonix, Evanston, IL, USA) at the SER-CAT beam line (22-ID) at the Advanced Photon Source synchrotron at Argonne National Labs. Prior to mounting, crystals were flash-frozen in a nitrogen stream (100 K) [29]. Crystals diffracted to 1.3 Å, and data up to 1.5 Å were used to solve the structure. Data reduction and scaling were carried out using HKL2000 [30]. Data collection procedures and statistics are shown in Table 1.

### 2.4. Structure Solution by Molecular Replacement

Molecular replacement was carried out using AMoRE [31] from the CCP4 program suite [32]. Initial phases were obtained by using a homology model of MppA based on the structure of *Salmonella enterica serovar Typhimurium* OppA (PDB ID: 1RKM) as a search model because the ligand-bound form of MppA was not yet available at the time this MppA structure was solved. MppA has a 46% amino acid sequence identity to OppA. The sequence alignment is shown in Supplementary Figure 1. A clear solution was not obtained when the entire homology model was used for molecular replacement. Because the structure of OppA is bilobed, the two lobes of the homology model were used separately as molecular replacement search models. Fragment I consisted of residues 23–284 and 509–537, and fragment II consisted of residues 288–501. Residues 285–287 and 502–508, which were predicted to belong to a linker region between the two lobes, were left out of the search model. Clear solutions were obtained only for fragment I, residues 23–284, and 509–537. Searches with fragment II did not yield a clear solution, even in cross-rotation calculations. The final molecular replacement solution consisted of coordinates for two copies of fragment I, one for each of the two molecules of MppA in the asymmetric unit. 

### 2.5. Structure Refinement

Five percent of the data was set aside for *R*_free_ calculations [33,34]. The two top solutions obtained after the translation function were further adjusted in AMoRE [31] before building an initial model. This model still lacked most of the amino acid residues in domain 2 of both molecules in the asymmetric unit. The initial model was rebuilt during several rounds of automated refinement using the program ARP/wARP [35], followed by manual building of the missing amino acids. This step was aided by the superposition of the homology model of MppA described above onto the partially built structure. The resulting model of MppA was refined using Refmac5 [36]. Additional refinement consisted of alternating rounds of positional refinement with the CNS program package [37] and manual fitting of the model to electron density maps using the programs O and Coot [38,39]. Ordered water molecules were added to the model based on electron density from composite simulated annealing omit maps; geometry, and refined B-factors. Statistics of model refinement are listed in Table 1. Model validation was performed using the Protein Data Bank [40,41] validation service including MolProbity [42]. Coordinates for the structure of MppA from *E. coli* were deposited in the Protein Data Bank [40,41] with the Database ID 4TOZ.

## 3. Results

### 3.1. Expression, Purification, Crystallization, and Structure Solution of MppA

MppA with a C-terminal hexahistidine tag was expressed as described in the methods section. Up to 10 mg/mL of pure protein was obtained from a 1 L culture after purification by affinity chromatography and ammonium sulfate precipitation. Crystals in space group P2_1_ diffracted to 1.3 Å, and data up to 1.5 Å were used for data collection. A homology model based on *Salmonella enterica serovar Typhimurium* OppA was used as a molecular replacement model. The final model has an R_free_ [33,34] of 20.5 and Rfactor of 0.18. There are two molecules of MppA in the asymmetric unit. As observed with most *E. coli* PBPs, MppA is processed after amino acid number 22 during its maturation, and thus the structure begins at amino acid 23. Molecule A consists of amino acid residues 23–537, and molecule B consists of amino acid residues 26–540. Amino acid residues 538–540 in molecule A and amino acid residues 23–25 in molecule B were disordered and were left out of the final model. There is only one amino acid that is an outlier in the Ramachandran plot, Ala46 in chain A, which is found within a loop on the protein surface. The electron density for this amino acid is weaker than for surrounding amino acids but is still visible. Amino acids Ser538, Arg539, and His540 in molecule B are not found in the wild-type protein but are extra amino acids encoded by codons near the cloning site of the expression vector, pQE60. The alpha carbons of the two monomers were overlaid using PyMol [43] with a root mean square deviation (RMSD) of 501 alpha carbons of 0.86 Å. When only domain 1 was overlaid, the RMSD was 0.18 for 237 alpha carbons, and the RMSD was 0.19 in domain 2 for 198 alpha carbons, which indicates that the two domains are very similar in structure in the two monomers, but one monomer is slightly more open than the other.

In the refined structure, there is a clear electron density for a glutamate at position 216 instead of an aspartate, and an aspartate is located at position 359 instead of a glutamate in both of the molecules in the asymmetric unit. We do not know if these sequence differences are the result of mutations during cloning or due to differences in the original reported sequence of MppA.

### 3.2. Overall Structure of MppA

**Figure 1:**
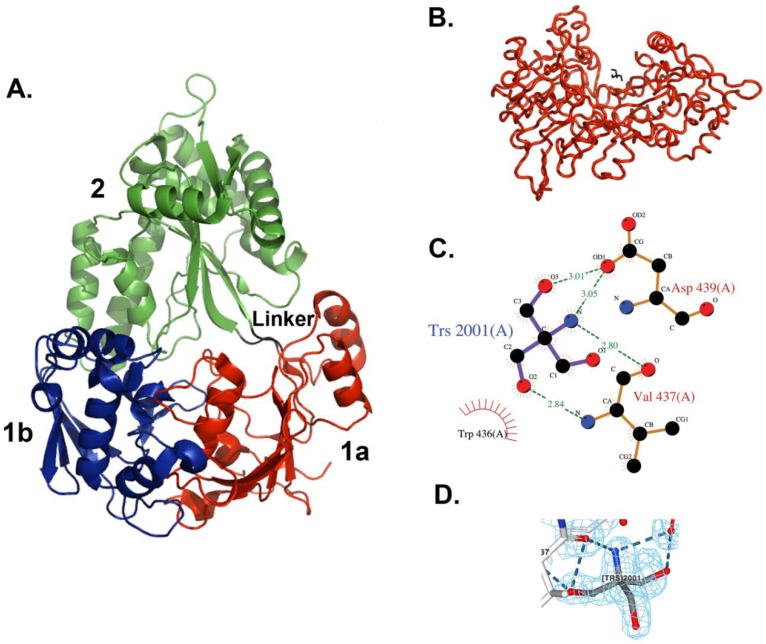
X-ray crystal structure of *E. coli* MppA. (**A**) The open form of MppA is a pear-shaped, bilobed structure. Domain 1a is shown in red, 1b is in blue, and 2 is in green. The linker region is in black. (**B**) The Tris binding site. Contacts are made between a bound Tris molecule (black bonds) with amino acid residues in one copy of MppA (red, molecule A) in the asymmetric unit. (**A**,**B** were made using PyMol [43]). (**C**) The Tris buffer molecule interacts with the side chain of Asp439 and the backbone of Val437, near the sidechain of Trp436. (**C** was made with LigPlot within Procheck). (**D**) 2fo-fc Electron density map around bound Tris molecule contoured at 1.0 (made with NGL viewer [46,47]).

The structure of MppA is bilobed, which is similar to that of OppA and other proteins in this family (Figure 1). The two domains have been designated as domains 1 and 2. Domain 1 is made up of two smaller domains, 1a (residues 23–65, 197–285, and 509–540) and domain 1b (residues 66–196). Domain 2 consists of residues 288–506. Domain 1a contains six beta strands, two short alpha helices, and eleven loops. Domain 1b contains six beta strands, four helices, two short helices, and thirteen loops. Domain 2 contains five beta strands, nine helices, and thirteen loops arranged as an alpha–beta–alpha sandwich. Residues 286–287 and 507–508 make up the hinge region between the two lobes. There are only slight variations in the structures of the two molecules in the asymmetric unit. Residues 23–31 and 536–540 at the N- and C-termini of the two molecules have different conformations. A search for proteins with similar structures in the Protein Data Bank [40,41] was performed using the program Dali [44]. As expected, the best scoring matches, with Z scores above 25, were other periplasmic ligand-binding proteins, including several oligopeptide binding proteins, dipeptide binding proteins, nickel binding proteins, heme binding proteins, and chitin binding proteins. The highest structural similarity is with the ligand-free form of *E. coli* OppA [45], an oligopeptide-binding protein (PDB ID = 3TCH), with a Z score of 51.4 and superposing 515 equivalent C-alpha atoms with an RMSD of 1.6 Å. The structural similarity is along the entire length of the protein. A superposition of the alpha carbons in only domain 1 yields an RMSD of 0.67 for 232 alpha carbon positions, and for domain 2 an RMSD of 0.76 for 192 alpha carbon positions.

### 3.3. Tris Binding Site

In the new crystal structure, no peptide was found bound to the protein, but subunit A has a molecule of Tris buffer bound on the surface, whereas molecule B does not. The Tris molecule makes direct hydrogen bonds with two amino acid residues in domain 2, Val437, and Asp439 (Figure 1).

## 4. Discussion

### Comparison to the Ligand-Bound Conformation

**Figure 2:**
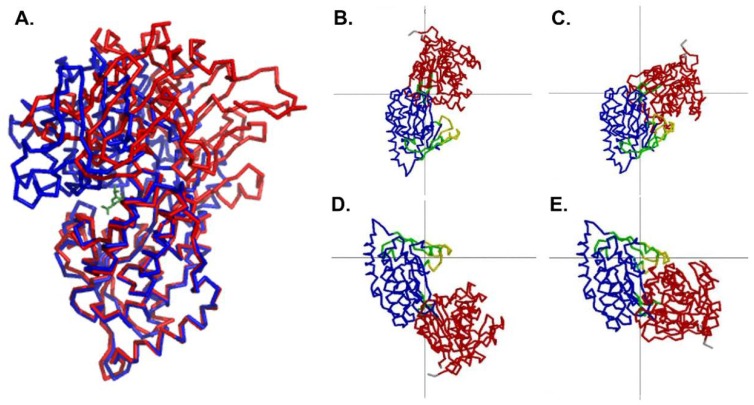
Comparison of the open conformation and the closed conformation. (**A**) Domain 2 of the open conformation of MppA (PDB ID 4TOZ, red bonds) was superposed on the equivalent alpha carbons of the closed conformation (PDB ID 3O9P, blue bonds). The murien tripeptide (green bonds) is shown bound to the closed conformation of MppA. (Superposition was calculated using Coot [39]. Figure made using PyMol [43]). (**B**–**E**) show the results of an analysis of domain motions by DynDom [48]. (**B**,**C**) focus on the larger conformational change with domain 2 (blue) fixed in position and domain 1 (red) moving relative to it. The green residues near the center of the figure are the bending residues, and the grey lines cross at the center of the rotation. (**D**,**E**) focus on the smaller conformational change that takes place within domain 2, with the larger subdomain (blue) fixed and the smaller subdomain (yellow) moving relative to it. The grey lines cross at the center of rotation, and the green residues near the center of rotation are the bending residues.

The conformation of the unliganded MppA protein was compared to that of the liganded protein (PDB ID 3O9P) using DynDom [48]. Upon ligand binding, the large domain undergoes 50.4° rotation and 0.4 Å translation relative to the small domain when comparing the ligand-bound form to monomer A in the asymmetric unit. In contrast, the large domain undergoes 53.4° rotation and 0.3 Å translation when compared to monomer B upon ligand binding. The bending occurs in amino acid residues 286–291 and 505–509. A least squares superposition of the structures based on the alpha carbons of domain 2 is shown in Figure 2. Similar rotation and translation of the large domain relative to the small domain are seen in other members of the PBP protein family such as the *E. coli* dipeptide binding protein (PDB IDs 1DPP [16] and 1DPE [17], 53.7° rotation and 0.9 Å translation) and the *S. typhimurium* lysine/arginine/ornithine binding protein (PDB IDs 1LST [13], 2LAO [13], 51.5° rotation and 0.4 Å translation). Less movement of the domains is observed when comparing the open and closed structures of the *Salmonella enterica serovar Typhimurium* oligopeptide binding protein (PDB IDs 1OLA [14], 1RKM [15], 16.3° rotation and 0 Å translation), possibly due to crystal contacts affecting the extent of opening in the unliganded form.

There is also a smaller change in conformation within Domain 2 of MppA. Residues 289–383, 393–411, and 426–506 move relative to residues 384–392 and 412–425. The bending residues are 380–384, 392–393, 402–412, and 422–426. This involves a 17.7 degree rotation of the two groups of amino acids, with no translation. Figure 2 illustrates the movements of the domains as calculated by DynDom.

**Figure 3:**
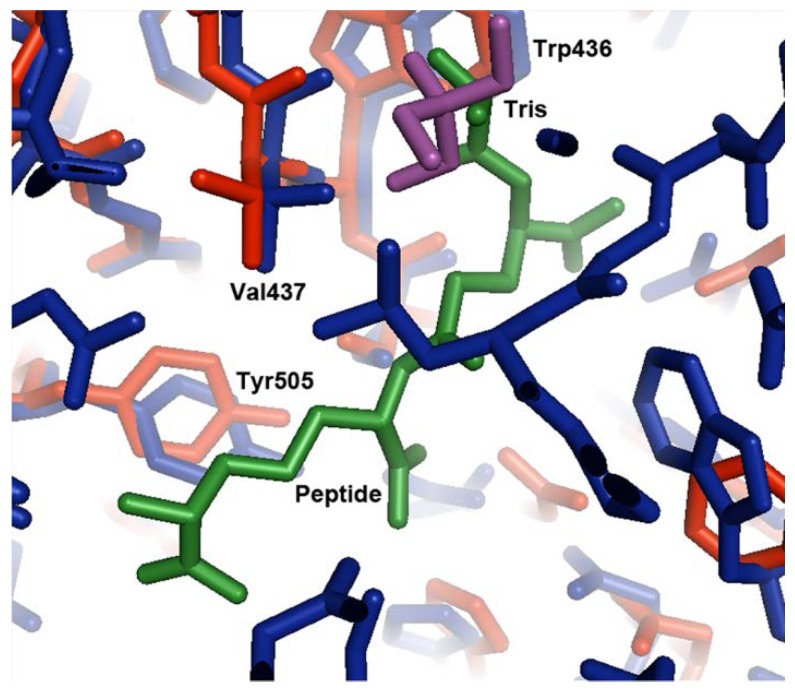
Comparison of the Tris binding site in the open conformation to the tripeptide binding site in the closed conformation. Domain 2 of the open conformation of MppA (PDB ID 4TOZ, red bonds) was superposed on the equivalent alpha carbons of the closed conformation (PDB ID 3O9P, blue bonds). The Tris buffer molecule (purple bonds) superposes near the L-alanyl moiety of the tripeptide (green bonds).

In the superposed structures, the Tris molecule found in the open form of the protein is located in a similar position to atoms N1, C2, C1, C3, and O1 of the L-alanine moiety of the bound Mtp peptide ligand in the ligand-bound structure (Figure 3). The amino group of the Tris molecule forms hydrogen bonds to the sidechain of Asp439 and the backbone carbonyl group of Val437 in domain 2. The O2 hydroxyl group forms a hydrogen bond to the nitrogen of Val437. The other two hydroxyl groups form hydrogen bonds to ordered water molecules. In the peptide bound structure, the free amino group of the alanyl residue in the tripeptide also forms hydrogen bonds with the sidechain of Asp439 and the backbone carbonyl group of Val437. The carbonyl group of the alanyl residue forms a hydrogen bond to the backbone nitrogen of Val437. Although the interactions between MppA and the Tris molecule and MppA and the alanyl moiety of the peptide are similar, the Tris molecule does not make interactions with domain 1 of MppA; therefore, it does not promote the formation of the closed conformation of the protein. In contrast, the other residues in the peptide ligand make interactions with domain 1 of MppA; therefore, the bound peptide promotes the formation of the closed conformation of MppA.

## 5. Conclusions

We have solved a high-resolution structure of MppA in the open conformation. Now, crystal structures are available for this member of the PBP protein family in both the liganded/closed form and the unliganded/open form. In addition, we have added MppA to the possible PBP targets for protein engineering.

## Figures and Tables

**Table 1:** Data Collection and Refinement Statistics for 4TOZ.

*A. Data statistics*		
Resolution	50–1.5 Å	
Space group	P2_1_	
Unit cell dimensions	a = 74.97, b = 76.81, c = 89.57, β = 91.58	
Number of unique reflections	161,107	
Completeness	99.6%	
I/σ(I)	6.82	
*B. Refinement*		
Resolution range (refinement)	35–1.5 Å	
Crystallographic *R*-factor ^a^ (*R*_work_)	0.18	
Free *R*-factor ^b^ (*R*_free_)	0.205	
Number per asymmetric unit		
MppA molecules	2	
Tris molecules	1	
Protein atoms	8150	
Solvent atoms	1647	
Deviation from standard geometry (Gfactors ^c^)		
Dihedral angles		
Phi-psi distribution	−0.10	
Chi1-chi2 distribution	0.12	
Chi1 only	0.19	
Chi3 and Chi4	0.59	
Omega	0.54	
Main-chain covalent forces		
Main-chain bond lengths	0.68	
Main-chain bond angles	0.45	
Average B value (Å^2^)		
All atoms	16.1	
of protein	13.7	
of Tris ligand	26.1	
of waters	28.0	
Ramachandran plot statistics from ProCheck		
Number of residues Percent		
Most favored regions	817	90.5
Additional allowed regions	84	9.3
Generously allowed regions	1	0.1
Disallowed regions	1	0.1

^a^*R*-factor = ∑|*Fo*| − |*Fc*|/∑|*Fo*|, where *Fo* and *Fc* are the observed and calculated structure factor amplitudes, respectively. ^b^ *R*_free_ is the *R*-factor calculated, with 5% of the reflections chosen at random and omitted from refinement [33,34]. ^c^ *G*-factors provide a measure of how out-of-the-ordinary a property is. Values below −0.5 are unusual, and values below −1.0 are highly unusual.

## Supplementary Materials

The following are available online at http://www.mdpi.com/2079-7737/7/2/30/s1, Figure S1: Sequence alignment of MppA and OppA made with Esprit.
