# Supplementary material for: Open Conformation of the Escherichia coli Periplasmic Murein Tripeptide Binding Protein, MppA, at High Resolution
Source: Biology (Basel). 2018 May 19;7(2):30. doi: 10.3390/biology7020030 (PMC6022919; doi:10.3390/biology7020030)
Supplement: Supplementary file 1 [file biology-07-00030-s001.pdf]

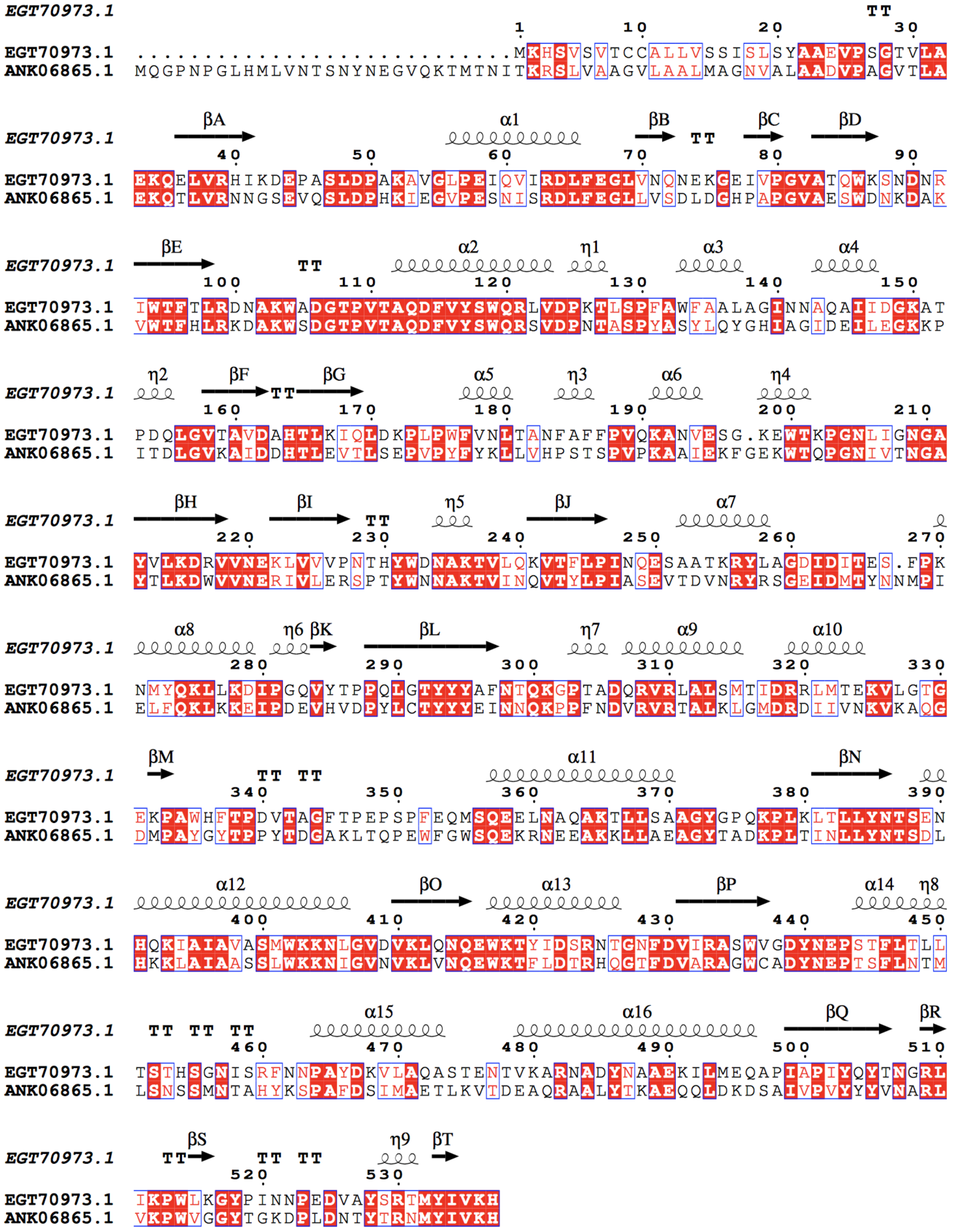

Figure S1. Sequence alignment of MppA and OppA made with Esprit [1].

Reference

1. Robert, X.; Gouet, P. Deciphering key features in protein structures with the new ENDscript server. *Nucleic Acids Res* **2014**, *42*, doi:10.1093/nar/gku316.
